# The prevalence of work-related stress, and its association with self-perceived health and sick-leave, in a population of employed Swedish women

**DOI:** 10.1186/1471-2458-9-73

**Published:** 2009-03-02

**Authors:** Kristina Holmgren, Synneve Dahlin-Ivanoff, Cecilia Björkelund, Gunnel Hensing

**Affiliations:** 1 Department of Clinical Neuroscience and Rehabilitation/Occupational Therapy, Sahlgrenska Academy at the University of Gothenburg, Göteborg, Sweden; 2 Department of Public Health and Community Medicine/Primary Health Care, Sahlgrenska Academy at the University of Gothenburg, Göteborg, Sweden; 3 Department of Public Health and Community Medicine/Social Medicine, Sahlgrenska Academy at the University of Gothenburg, Göteborg, Sweden

## Abstract

**Background:**

Women report more occupational ill-health and are more sick-listed than men. Exploration of women's working conditions would therefore seem to be valuable. In this study we investigated the prevalence of work-related stress and its association with self-perceived health and sick-leave in a population of employed, working Swedish women.

**Methods:**

This cross-sectional population study comprised 424 employed, working women who answered questionnaires on work-related stress, self-perceived health and sick-leave. The odds ratio (OR) with 95% confidence interval (CI) was calculated in order to analyse the association between the exposure variables of work-related stress and outcome variables of ill-health symptoms, self-rated health and sick-leave.

**Results:**

Ten percent of the group reported high *perceived stress owing to indistinct organisation and conflicts*, and 25% high *perceived stress owing to individual demands and commitment*. Twenty-two percent reported low *influence at work *and 33% reported work interference with leisure time. All categories of overall work-related stress were significantly associated with increased odds of high level of illness symptoms, with the highest OR for high *perceived stress owing to indistinct organisation and conflicts *and high *perceived stress owing to individual demands and commitment *with an OR of 3.17 (CI = 1.51–6.62) and 4.53 (CI = 2.71–7.56) respectively. High *perceived stress owing to indistinct organisation and conflicts *and low *influence at work *were significantly associated with sick-leave with an OR of 3.85 (CI = 1.59–9.30) and 2.54 (CI = 1.17–5.48) respectively.

**Conclusion:**

This study showed an association between, on the one hand, work-related stress, and on the other hand, illness symptoms and sick-leave. Distinguishing between the occurrence of negative work characteristics, and the immediate perception of stress because of these, resulted in a broad view of women's working conditions and expanded knowledge of work-related stress in women.

## Background

Work characteristics such as insufficient leadership, injustice at work and poor organisational climate have been linked to sickness absence in both women and men [1-3]. Furthermore, high demands, low control and low social support have, in several studies, been found to increase the risk of sickness absence and musculoskeletal and psychiatric disorders [4-7]. Increased workload with perceived high psychological and physical demands has been connected to symptoms of illness, as well as to sick-leave, and this is more pronounced in women [6,6-9]. Physical and mental demands greater than own capacity and high work stress combined with lack of control over working hours constituted a risk of sick-leave among women [8,10]. Person-related characteristics, such as over-commitment, strenuous effort at work and low self-efficacy, have also been associated with poor health perception and sick-leave [11,12]. Even though we know that different work-related factors influence health and sick-leave outcomes, little research has focused on the prevalence of work-related stress in a general working population of women.

During the 1990s the rate of sick-leave increased dramatically in Sweden, but has somewhat decreased in the last few years. The costs for early retirement pensions, however, have increased and over half a million people are on disability pension. Women account for the majority of sick-leave as well as early retirement pension [13,14]. In just a few years the proportion of psychiatric sickness certification diagnoses has risen from 18% to over 30% on sickness certificates. Diagnoses of depression, stress reactions and anxiety syndromes show the greatest increase [13,14]. Large reductions in private as well as public funding have resulted in increased workload, with a greater risk of sick-leave [14,15]. In the same period of time the psychosocial work environment has deteriorated, i.e. stressful work, work demands and work pace have increased, and the effect is especially pronounced in women [13,14]. Women also report a decline in health with more fatigue and musculoskeletal pain than men [14]. In Sweden, women and men are to a large extent concentrated in different sectors of the labour market [16]. They might therefore be exposed in different ways and to different degrees to work-related stress. Consequently, it is important to explore women's working situation further.

In a qualitative, explorative study [17] women on sick-leave owing to work-related strain described the gradual road to sickness absence as a process going from controlling everyday life, to total loss of control of working and private life. At work, both environmental and personal factors contributed to the process. The women described the work situation as strained, and they suffered from lack of organisational clarity, little influence and unsolved conflicts. The participants saw themselves as people with high demands on their capacity, strong sense of responsibility and having difficulties setting limits. This combination of work-related factors and personal characteristics led to loss of control and sick-leave. Based on that study a questionnaire was developed with the purpose of assessing the perceived stress dimension in relation to these environmental and personal factors [18]. To determine the need for preventative steps decreasing the development of sick-leave in women it is of some importance to find out how common work-related stress is in a general population of women, and to obtain more knowledge about the relationship between women's work-related stress and their health perception and sick-leave. To our knowledge, there are few studies on the prevalence of work-related stress in women. The aim of the present study was to investigate the prevalence of work-related stress and its association with self-perceived health and sick-leave in a general population of employed, working women aged thirty-eight or fifty.

## Methods

### Research design and population

This cross-sectional study of women aged thirty-eight and fifty was part of a longitudinal population based study – 'The Population Study of Women in Gothenburg, Sweden'. The two-cohort design has been aimed to capture two important stages in women's life. Cohort comparisons have been carried out in 1968–69, 1980–81, 1992–93 and 2004–05 [19]. In the latest study, stretching from October 2004 to April 2005, a random sample of 38-year-old and 50-year-old women registered for census purposes in Gothenburg was identified and invited to participate in a free health examination. Additionally, 85 participants aged 38 in the 1992–93 cohort, and who in 2004–05 were 50 years old, were invited. In all, 846 women were asked to participate (7 were excluded owing to difficulties in speaking and understanding Swedish) (Table 1). The external drop-outs were 339, of whom 191 declined or did not turn up and 148 could not be contacted (participation rates 60% and 58% respectively). In total, 500 women accepted and participated in the study (Table 1) [20].

**Table 1:** Population procedure of 'The Population Study of Women in Gothenburg, Sweden' and the present cross-sectional study in 2004 to 2005.

	Invited	Excluded	External drop-outs	Participants	Participation rate	Eligible for present study	Internal drop-outs	Participants in present study
	n	n	n	n	(%)	n	n	n
Total cohort	846	7	339	500	59	433	9	424
	
38-years-old	343	5	131	207 (41%)	60	177	5	172 (41%)
50-years-old	503	2	208	293 (59%)	58	256	4	252 (59%)

Inclusion criteria for the present cross-sectional study were employed or self-employed women. Four hundred and thirty-three women of the sample fulfilled these criteria. Of these, nine women did not complete the work stress questionnaire and dropped out. In all, 424 women participated in the present study (Table 1). The study was approved by the Ethics Committee, University of Gothenburg, Sweden.

### Procedure

The participants went through a half-day health examination which included an interview, questionnaires, physical examinations, measurements and blood tests [20]. At the end of the health examination all participants received a questionnaire designed for the present study and a stamped addressed envelope in which to return the completed questionnaire. Two reminders were given by telephone at two-week intervals.

### Exposure variables

#### Work characteristics and perceived stress

The instrument used was the Work Stress Questionnaire, developed from the qualitative study described above [17,18]. Two main themes were identified. One was related to factors at work and the other to the persons themselves. Categories were recognized and questions were constructed. The self-assessed instrument consists of 21 main questions grouped into four categories: *indistinct organisation and conflicts *and *individual demands and commitment *contain 7 questions respectively and answers to these questions are Yes, Partly and No; *influence at work *and *work interference with leisure time *contain 4 and 3 questions respectively and answers are given on a four-point ordinal scale – Yes, always, Yes, rather often, No, seldom and No, never. Each of the questions in the categories *indistinct organisation and conflicts *and *individual demands and commitment *has an appended question 'Do you perceive it as stressful?' Answers to this question are given on a four-point ordinal scale – Not at all stressful, Less stressful, Stressful and Very stressful. These were grouped into two categories of perceived stress owing to work characteristics: *perceived stress owing to indistinct organisation and conflicts *(7 questions) and *perceived stress owing to individual demands and commitment *(7 questions). The reliability of the questionnaire was tested in a test-retest study by a non-parametric statistical method for evaluation of paired data. The test values were close to zero throughout, which indicates a high level of reliability [18]. The items were evaluated by a pilot group, representing the questionnaire's target group, who agreed to face validity of the questions and their content [18].

#### Definition of overall work-related stress

Overall work-related stress was defined as follows: Confirmatory answers of the items within the category *indistinct organisation *and *conflicts *and *individual demands and commitment *were counted for every participant. In order to find enough exposure differences, without having to compare the extremes, we chose to dichotomise at the upper quartile. For *indistinct organisation and conflicts *(7 questions), high level of exposure was defined as confirmatory answers to 4–7 items and low level of exposure was defined as confirmatory answers to 0–3 items. For *individual demands and commitment *(7 questions), the cut-off for high level was 7 items and low level 0–6 items. Each participant's median response category of the four questions in *influence at work *was calculated and then dichotomised into high influence (always or often) and low (never or seldom). The median response category of the three questions in *work interference with leisure time *for each participant was calculated and then dichotomised into low (never or seldom) and high (always or often). Further, each participant's median response category of the seven questions respectively in *perceived stress owing to indistinct organisation and conflicts *and in *perceived stress owing to individual demands and commitment *was calculated and then dichotomised into low stress perception (defined as no confirmation of perceived stress, not stressful or less stressful), and high stress perception (confirmatory answers to stressful or very stressful).

#### Test of validity

The exposure variables as defined above were validated against a question about general experienced stress. This was the only question about stress in the study of 'The Population Study of Women in Gothenburg, Sweden' and had earlier been used in these longitudinal surveys [19]. The question was: 'Have you experienced any period of stress during a longer period of time, i.e. a month or more? The word stress implies that you have been irritable, tense, nervous, anxious or sleepless in relation to work, health, family or in relation to conflicts in these areas or in relation to something else'. Six possible statements were given on an ordinal scale: have never experienced any period of stress, have experienced period of stress but not the last 5 years, have experienced period of stress the last 5 years, have experienced several periods of stress the last 5 years, lived with constant stress the last year, lived with constant stress the last 5 years. The answers were dichotomised into low stress experience ('have never experienced stress' to 'have experienced a period of stress in the last 5 years) and high stress experience ('have experienced several periods of stress' to 'lived with constant stress for the last 5 years').

The six categories of work-related stress were all significantly associated with the question about general experienced of stress. The highest OR were high *perceived stress owing to indistinct organisation and conflicts *and high *perceived stress owing to individual demands and commitment *with an OR of 4.16 (1.92–9.00) and 3.98 (2.42–6.54), respectively – not presented in any table.

### Outcome variables

#### Self-rated symptoms

Self-rated symptoms were assessed by one question: 'Have you, during the last 3 months, been troubled by any of the symptoms listed?' Thirty different mental and physical symptoms were listed and two possible statements were given: Yes or No. Each participant's number of stated symptoms was counted and dichotomised according to median cut: high level of self-rated symptoms was defined as 8 or more stated symptoms. This question has been used in the longitudinal survey of 'The Population Study of Women in Gothenburg, Sweden' [19,21].

#### Self-rated health

Self-rated health was assessed by the statement: 'In general, would you state your health as being ...'. Answers were given on a five-point ordinal scale – Excellent, Very good, Good, Fair and Bad. Self-rated health has been shown to be a good indicator of health, predicting morbidity and mortality in prospective studies [22]. Poor self-rated health was dichotomised into poor (Fair/Bad) and good (Excellent/Very good/Good). Since only a small group (n = 48) assessed poor self-rated health, we chose to include a salutogenic perspective and assessed the associations with good self-rated health, which was defined and dichotomised into good (Excellent/Very good) and poor (Good/Fair/Bad).

#### Sick-leave

Self-reported sick-leave was assessed by the questions: 'Are you on sick-leave at the moment?', 'To what degree?' and 'For how long have you been sick-listed? State the number of weeks'. These questions were not validated. Voss et al [23] found, however, the agreement between self-reported and registered data on sick-leave good.

### Statistics

Descriptive statistics of work-related stress were calculated. The chi-squared and Fisher's two-tailed exact test were used to test differences in the proportions between groups; age-group (38-year-old/50-year-old), educational level (>12 years/10–12 years, ≤ 9 years), occupational class (manager, high and middle level non-manual/low level non-manual, manual), employer (private, self-employed, combined/public) and sick-leave/no sick-leave. The odds ratio (OR) with 95% confidence intervals (CI) was calculated in order to analyse the association between the exposure variables of work-related stress and outcome variables of sick-leave, self-rated health and symptoms of illness. The OR was also used to validate the exposure variable against the question about general experienced stress. The logistic regression models were used to adjust for age-group, educational level, occupational class and employer.

## Results

### Demography and distribution of exposure differences

A significantly larger proportion of the 50-year-old women were public employees (p < 0.001). A majority had professions characterised as high or middle level non-manual occupation. Thirty participants were on sick-leave and sick-leave spells had a range from less than 1 month to over 4 years. Of those on full-time sick-leave all but one had been on sick-leave <2 months. A significantly larger proportion of those on sick-leave belonged to the age group of 50-year-olds (p < 0.006) (Table 2).

**Table 2:** Employment and educational data, sick-leave and partial disability pension of the participants.

	Entire group n = 424		38-years-old n = 172		50-years-old n = 252		
	n	%	n	%	n	%	P
Employment							0.186
Permanent	364	86	143	83	221	88	
Temporary (deputy)	60	14	29	17	31	12	
Hours worked/week							0.228
Full-time (>32)	314	74	132	77	182	72	
Part-time (<32)	102	24	36	21	66	26	
Missing data	8	2	4	2	4	2	
Employer							0.001¤
Public	227	53	69	40	158	63	
Private	138	33	78	45	60	24	
Self-employed	32	8	15	9	17	7	
Combined	27	6	10	6	17	7	
Occupational class							0.538
Manager	26	6	12	7	14	5	
High level non-manual	131	31	46	27	85	34	
Middle level non-manual	98	23	45	26	53	21	
Low level non-manual	130	31	53	30	77	31	
Manual	20	5	8	5	12	5	
Missing data	19	4	8	5	11	4	
Educational level							0.073
> 12 years	233	55	93	54	140	57	
10 – 12 years	149	35	68	40	81	32	
<= 9 years	42	10	11	6	31	11	
							
Sick-leave	30	7	5	3	25	10	0.006¤
Partial disability pension	18	4	5	3	13	5	0.814

¤Statistically significant

The two age-groups are presented separately and statistical significance levels of differences between the groups are given (n = 424).

No differences were found between age groups concerning exposure to work-related stress. The proportion who reported a high level of *work interference with leisure time *was, however, significantly larger among individuals with a high education level (p = 0.03), higher occupational class (p = 0.03) and private employment (p = 0.04). The proportion of high *indistinct organisation and conflicts *was larger among public employees (p = 0.01) and high *individual demands and commitment *was larger among higher occupational class (p = 0.01). A larger proportion among those on sick-leave reported low *influence at work *(p = 0.02) and high *perceived stress owing to indistinct organisation and conflicts *(p = 0.01) – data not shown.

### Prevalence of work characteristics and perceived stress

The prevalence of the three most reported work characteristics in *indistinct organisation and conflicts *was increased work-load (66%), conflicts at work (65%) and involved in conflicts at work (35%). The three most reported characteristics in *individual demands and commitment *were putting high demands on oneself at work, being engaged in one's work and thinking about work after the working day with 93, 94 and 92% respectively. In *influence at work *29% reported difficulties in influencing decisions and 25% reported difficulties in deciding the working pace. The prevalence of work characteristics in *work interference with leisure time *was approximately 30% for the three items (Table 3).

**Table 3:** Prevalence of work characteristics in the cohort of 38 and 50-year-old Swedish women (n = 424).

Indistinct organisation and conflicts	% (95% CI)	n
Increased work-load	66 (62 – 71)	281
Unclear goals at workplace	34 (29 – 38)	143
Unclear work assignments	14 (10 – 17)	56
Unclear leadership	14 (11 – 18)	60
Conflicts at work	65 (60 – 69)	275
Involved in conflicts at work	35 (60--41)	97¤
Supervisor not solving conflicts	63 (57 – 69)	173¤

Individual demands and commitment		

High demands on oneself at work	93 (90 – 95)	395
Engaged in one's work	94 (91 – 96)	397
Think about work after working-day	92 (88 – 94)	388
Hard to set limits	87 (84 – 90)	371
High responsibility for one's work	59 (54 – 63)	249
Work over-time	71 (66 – 75)	301
Sleep disturbance on account of work	43 (38 – 48)	181

Influence at work		n

Time to finish assignments	90 (86 – 92)	380
Influence decisions at work	71 (66 – 75)	300
Supervisor consider your views	84 (81 – 88)	348*
Deciding on working pace	75 (71 – 79)	318

Work interference with leisure time		

Hard to find time to see the nearest	28 (24 – 32)	118
Hard to find time to see friends	34 (29 – 39)	144
Hard to find time for recreational activities	36 (32 – 41)	154

¤ 149 persons did not have any conflicts at work

* 12 persons did not answer because of not having a supervisor.

The prevalence of the three most reported items in *perceived stress owing to indistinct organisation and conflicts *was 38% for increased workload, 23% for conflicts at work and 17% for reporting stress because of supervisors not solving the conflicts. Most prevalent items in *perceived stress owing to individual demands and commitment *were stress owing to hard to set limits with a prevalence of 44%, high demands on oneself at work, 29% and high responsibility for one's work, 29% (Table 4).

**Table 4:** Prevalence of perceived stress owing to the items in Indistinct organisation and conflicts and in Individual demands and commitment in the cohort of 38 and 50-year-old Swedish women (n = 424).

Perceived stress (PS) owing to the following items in Indistinct organisation and conflicts	High stress perception		No/Low stress perception	
	% (95% CI)	n	% (95% CI)	n
PS owing to increased work-load	38 (33 – 43)	160	62 (57 – 67)	264
PS owing to unclear goals at workplace	14 (11 – 18)	60	86 (82 – 89)	364
PS owing to unclear work assignments	7 (4 – 9)	28	93 (91 – 95)	396
PS owing to unclear leadership	3 (1 – 5)	12	97 (95 – 99)	412
PS owing to conflicts at work	23 (19 – 27)	98	77 (73 – 81)	326
PS owing to involved in conflicts at work	12 (9 – 15)	49	88 (85 – 91)	375
PS owing to supervisor not solved the conflicts	17 (13 – 21)	71	83 (79 – 87)	353

Perceived stress (PS) owing to the following items in Individual demands and commitment				

PS owing to high demands on oneself at work	29 (25 – 34)	125	71 (66 – 75)	299
PS owing to engaged in one's work	17 (14 – 21)	72	83 (79 – 86)	352
PS owing to think about work after working-day	25 (21 – 29)	106	75 (71 – 79)	318
PS owing to hard to set limits	44 (39 – 49)	187	56 (51 – 61)	237
PS owing to high responsibility for one's work	29 (25 – 34)	123	71 (66 – 75)	301
PS owing to work over-time/stress	24 (20 – 28)	102	76 (72 – 80)	322
PS owing to sleep disturbance on account of work	26 (22 – 31)	112	74 (69 – 78)	312

### Prevalence of overall work-related stress

The overall work-related stress was, as described in the method section, grouped into four categories of work characteristics and two categories of perceived stress owing to work characteristics. Twenty-nine percent reported a high level of exposure caused by *indistinct organisation and conflicts *and 26% a high level caused by *individual demands and commitment*. Ten percent of the entire group reported high *perceived stress owing to indistinct organisation and conflicts *and 25% high *perceived stress owing to individual demands and commitment*. Twenty-two percent stated low *Influence at work *and 33% *work interference with leisure time *(Table 5).

**Table 5:** The association between overall work-related stress and self-rated symptoms, poor and good self-rated health and sick leave, unadjusted and adjusted odds ratio# (indicated in bold) with 95% CI (n = 424).

Overall work-related stress	High level of self-rated symptoms (n = 224)	Poor (Fair/Bad) self-rated health (n = 48)	Good (Excellent/Very good) self-rated health (n = 222)	Sick-leave (n = 30)
% (n)	OR (95% CI) **Adjusted OR **(95%CI)	OR (95% CI) **Adjusted OR **(95%CI)	OR (95% CI) **Adjusted OR **(95%CI)	OR (95% CI) **Adjusted OR **(95%CI)
Indistinct Organisation and conflicts				
Low: 71 (300)	1.00	1.00	1.00	1.00
High: 29 (124)	1.63 (1.06 – 2.49)¤	1.85 (1.00 – 3.44)	0.72 (0.47 – 1.09)	1.44 (0.66 – 3.12)
	**1.70 **(1.10 – 2.64)¤	**1.89 **(1.00 – 3.59)	**0.70 **(0.45 – 1.10)	**1.51 **(0.68 – 3.38)
Individual demands and commitment				
Low: 74 (314)	1.00	1.00	1.00	1.00
High: 26 (110)	3.15 (1.96 – 5.06)¤	2.50 (1.34 – 4.62)¤	0.56 (0.36 – 0.86)¤	1.47 (0.67 – 3.25)
	**3.42 **(2.08 – 5.63)¤	**2.88 **(1.50 – 5.57)¤	**0.45 **(0.28 – 0.73)¤	**1.70 **(0.75 – 3.88)
Perceived stress due to indistinct organisation and conflicts				
Low: 90 (382)	1.00	1.00	1.00	1.00
High: 10 (42)	3.17 (1.51 – 6.62)¤	1.34 (0.53 – 3.37)	0.55 (0.36 – 0.73)¤	3.85 (1.59 – 9.30)¤
	**3.14 **(1.49 – 6.60)¤	**1.26 **(0.49 – 3.23)	**0.37 **(0.18 – 0.75)¤	**3.93 **(1.56 – 9.88)¤
Perceived stress due to individual demands and commitment				
Low: 75 (318)	1.00	1.00	1.00	1.00
High: 25 (106)	4.53 (2.71 – 7.56)¤	2.64 (1.42 – 4.91)¤	0.32 (0.20 – 0.51)¤	1.55 (0.70 – 3.43)
	**4.52 **(2.68 – 7.64)¤	**3.22 **(1.67 – 6.20)¤	**0.26 **(0.16 – 0.44)¤	**1.85 **(0.81 – 4.24)
Influence at work				
High: 78 (330)	1.00	1.00	1.00	1.00
Low: 22 (94)	1.89 (1.18 – 3.05)¤	1.70 (0.88 – 3.28)	0.45 (0.28 – 0.72)¤	2.54 (1.17 – 5.48)¤
	**1.86 **(1.14 – 3.04)¤	**1.55 **(0.78 – 3.05)	**0.46 **(0.28 – 0.77)¤	**2.37 **(1.06 – 5.30)¤
Work interference with leisure time				
Low: 67 (285)	1.00	1.00	1.00	1.00
High: 33 (139)	2.07 (1.36 – 3.15)¤	1.03 (0.55 – 1.95)	0.75 (0.50 – 1.13)	0.87 (0.39 – 1.95)
	**2.10 **(1.36 – 3.25)¤	**1.27 **(0.65 – 2.46)	**0.61 **(0.39 – 0.95)¤	**1.13 **(0.48 – 2.62)

¤ Statistically significant values of the confidence interval of OR

# Adjusted by age-group, educational level, occupational class and employer.

### Associations between overall work-related stress and outcome variables

All categories of work-related stress were significantly associated with increased odds of high level ill-health symptoms, with the highest OR for high *perceived stress owing to indistinct organisation and conflicts *and *perceived stress owing to individual demands and commitment *with an OR of 3.17 (1.51–6.62) and 4.53 (2.71–7.56), respectively. High *individual demands and commitment *and *perceived stress owing to individual demands and commitment *were significantly associated with Poor self-rated health with an OR of 2.50 (1.34–4.62) and 2.64 (1.42–4.91), respectively. The OR for high levels of exposure was low for those with a Good self-rated health. High *perceived stress owing to indistinct organisation and conflicts *and low *influence at work *was significantly associated with sick-leave with an OR of 3.85 (CI = 1.59–9.30) and 2.54 (CI = 1.17–5.48), respectively. After adjustments for age, education, occupation and employer, OR remained almost unaltered and significant in all categories (Table 5).

## Discussion

The aim of the present study was to investigate the prevalence of different types of work-related stress and its association with self-perceived health and sick-leave in a population of employed, working women aged thirty-eight or fifty years. The most common type of work-related stress was due to work interference with leisure time, followed by stress due to low influence at work, high perceived stress owing to indistinct organisation and conflicts and high perceived stress owing to individual demands and commitment. We also found a high prevalence of several specific work characteristics. Items in the category concerning *individual demands and commitment *showed the highest occurrence. The prevalence of *perceived stress *owing to certain work characteristics was, however, lower, although perceived stress owing to increased workload and hard-to-set limits had a prevalence of around 40% each. These findings of perceived stress can appear rather low in comparison with other studies, though. In a European report from 2000, 29% of female employees in Europe reported stress related to work [24]. One explanation of this disparity could be differences in measuring the exposure variables. The questionnaire in this study assesses not only the occurrence of work characteristics but also the immediate perception of the characteristics' stressfulness, i.e. if the characteristic is perceived as stressful or not. This study distinguishes between the occurrence of negative work characteristics and the perception of stress owing to these characteristics.

The European report also found that the prevalence varied between different occupations. Professionals reported highest stress, 40% compared with 17% in elementary occupations [24]. Two British studies, one of head teachers and one of police officers, found the prevalence of self-reported work-related stress to be 43 and 41% respectively [25,26]. We found few differences, however, between occupational classes, except for high *individual demands and commitment *where the prevalence was higher among the higher occupational classes. Furthermore, female head teachers reported more stress than male, and in both studies workload was a main stressor [25,26]. Correspondingly, in our study the prevalence of increased workload was high with high perceived stress as a result. Research finds that high workload constitutes a risk of ill-health perception and sickness absence [6-8,27].

It is notable that the prevalence of high *perceived stress *owing to individual demands and commitment was higher than for *perceived stress *owing to indistinct organisation and conflicts. Twenty-five percent reported high perceived stress owing to individual demands and commitment. High dedication to work and difficulties in managing the work situation seem to result in a high amount of stress. The issue is whether the occurrence of perceived stress will result in negative consequences or not. Some studies have linked over-commitment to a higher risk of poor health [11,28] and ill-health perception has been associated with sickness absence [29-31]. High effort and low reward, the so-called effort-reward imbalance, were also shown to have an adverse effect on self-reported health in a European comparative study [32]. In our earlier mentioned qualitative study, women sick-listed owing to work-related strain described putting high demands on themselves, having a high sense of responsibility and difficulty in setting limits as contributory factors in being put on sick-leave [17]. Perceived stress owing to individual demands and commitment may therefore have consequences for health outcomes and sick-leave.

### Work-related stress and associations with sick-leave

The prevalence of high influence at work was 78%. Likewise, the prevalence of low perceived stress owing to indistinct organisation and conflicts was as high as 90%. This was unexpected, since several studies point to a deterioration of work conditions, especially in women [13,14,24]. On the other hand, those reporting low influence at work and high perceived stress owing to indistinct organisation and conflicts had an increased probability of sick-leave, with an OR of 2.54 and 3.85 respectively. These connections correspond to several other findings linking low influence at work and work-related stress to an increased risk of sickness absence [1-4,9,31]. One might also have expected associations between the four other categories of work-related stress. To our knowledge, however, few studies have linked individual demands and commitment to sickness absence. Over-commitment has been related to vital exhaustion, low mental health and sleep disturbance, though [11,28,33]. The phenomenon of sickness absence is complex and needs to be understood in a broad context, on societal as well as organisational and individual levels [34,35].

### Work-related stress and associations with self-perceived health

In this study an association between on the one hand all the categories of overall work-related stress, and on the other hand a high level of self-rated symptoms was also found. Women reporting high *perceived stress owing to organisation and conflicts *and high *perceived stress owing to individual demands and commitment *had an increased probability of having a high level of self-rated symptoms with an OR of 3.17 and 4.53 respectively. This corresponds with earlier research where different work characteristics were associated with ill-health perceptions [6,7,11,28,29,31]. Correspondence between work-related stress and poor self-rated health was only found, however, in two of the six categories with the strongest association with *perceived stress owing to individual demands and commitments *having an OR of 2.64. Low reported work-related stress was, however, associated with good self-rated health. All of the significant associations between the exposure and outcome variables remained after adjustment for the confounders of age groups, educational level, occupational class, employer and sick-leave.

*Work interference with leisure time *was reported by 33% of the participants, and high level of interference was associated with a high level of self-rated symptoms having an OR of 2.07. Something to bear in mind is that women are part of the paid work force approximately to the same extent as men, with a participation rate of 80% to men's 86% [36]. At the same time unpaid work, such as household duties and childcare, has not decreased and women contribute the most to these chores [36,37]. In a study of white-collar women and men, women reported a higher total workload, including paid and unpaid work, more stress and higher severity of symptoms than men [38]. Work-family conflict has been found to constitute a risk of sickness absence in both women and men. It is most pronounced, however, and with poorer health outcomes, among women [39].

### Socio-demographic differences

Unexpectedly, we found few differences between the two age groups. A greater proportion of the 50-year-olds was sick-listed and in public employment, but no differences regarding exposure to work-related stress were shown. Some diversity was found among different occupational classes, educational levels and employment in relation to work-related stress exposure, though. *Work interference with leisure time *seems to be the category that differs the most between the groups. This is consistent with earlier findings where professionals perceive more stress than others [31] and also point out workload and work-family interference as main stressors [25]. More public than private employees found *indistinct organisation and conflicts *to be high. This may be explained by the large staff reductions and reorganisations in the public sector during the nineties, with higher workload for the retained staff as a consequence [14]. The lack of employment differences, i.e. between public and private employees regarding the exposure to work-related stress, could be explained by the occupational gender segregation which puts women in subordinate positions in the public as well as the private sector. In Sweden in general, three out of four managers are men; four out of five in the private sector [36]. Women appear to have less control at work than men and so-called active jobs (high control and high demands) constitute a risk for women, as opposed to men [9,29].

### Methodological considerations

This study had a cross-sectional design and no conclusions regarding causality can be made. Previous research, however, supports the finding that being exposed to work-related stress increases the risk of symptoms of illness and sick-leave [4,6-8,29]. Studies based on self report measures can be influenced by several factors, such as recall bias, socially desirable answers and exposure suspicion bias. It is therefore essential to interpret the results with caution. In this particular study we do not think that these possible sources of bias only follow one direction, i.e. towards overestimation. From clinical experience it is not uncommon that individuals exposed to severe stress underestimate the sources of stress in their lives. It is notable that some statistically significant values of the confidence interval of OR were wide and therefore the results have to be treated with caution. One possible limitation of the study was that the exposure variables were assessed with a recently-developed questionnaire. It has been found to have high reliability and face validity, but further research is required to ensure its validity. In this study, the validity was tested and correspondence was found between all categories of work-related stress and general stress experienced. Some of the items had a low prevalence and should perhaps have been considered for omission. The grouping of the items in different categories of work-related stress was, however, based on empirical findings in a qualitative study [17] and, since this questionnaire is still under development, we decided to let the items remain. Despite its limitations, the Work Stress Questionnaire has shown new ways of assessing work-related stress. The advantages with this questionnaire lie both in the design, which combines environmental and personal work characteristics, and in the quality of assessments of the experience of perceived stress in relation to each specific item.

As regards selection bias, the education level was higher (>12 years = 55%) in the study population than in the general population of women in Gothenburg in 2004 (>12 years = 44%) [40]. The study population, however, is not completely comparable with the general population, since only employed women were included. The general population also includes, for example, women who have not yet entered the labour market and unemployed women presumed to have a lower educational level. The sickness absence rate was somewhat higher in the study population (7%) than in the general female population of 2004 (5.6%) [36]. This can be explained by the higher representation of 50-year-olds.

## Conclusion

In order to devise preventative steps, it is important to identify individuals at risk of symptoms of illness and sickness absence owing to work-related stress. This study showed an association between on one hand work-related stress, and on the other, self-rated symptoms and sick-leave in employed, working women aged 38 or 50. By using the Work Stress Questionnaire that distinguishes between the occurrence of a negative work characteristic and the immediate perception of stress owing to the characteristic, we get a broader view of women's working conditions and an expanded knowledge of work-related stress in women. These findings imply that work-related stress among a general population of Swedish middle-aged employed women is an important public health issue.

## Competing interests

The authors declare that they have no competing interests.

## Authors' contributions

The work with this study was based on true collaboration between the authors. KH, SDI, GH contributed to the interpretation of data and in the development of the manuscript. KH, SDI, CB, GH have all contributed to the design of this study and revision of the manuscript. All authors read and approved of the final manuscript.

## Pre-publication history

The pre-publication history for this paper can be accessed here:


